# Secular humanism and "scientific psychiatry"

**DOI:** 10.1186/1747-5341-1-5

**Published:** 2006-04-25

**Authors:** Thomas Szasz

**Affiliations:** 1 Professor Emeritus of Psychiatry, SUNY Upstate Medical University, Syracuse, New York, US

## Abstract

The Council for Secular Humanism identifies Secular Humanism as a "way of thinking and living" committed to rejecting authoritarian beliefs and embracing "individual freedom and responsibility ... and cooperation." The paradigmatic practices of psychiatry are civil commitment and insanity defense, that is, depriving innocent persons of liberty and excusing guilty persons of their crimes: the consequences of both are confinement in institutions ostensibly devoted to the treatment of mental diseases. Black's Law Dictionary states: "Every confinement of the person is an 'imprisonment,' whether it be in a common prison, or in private house, or in the stocks, or even by forcibly detaining one in the public streets." Accordingly, I maintain that Secular Humanism is incompatible with the principles and practices of psychiatry.

My aim in this paper is to ask, is Secular Humanism compatible with so-called Scientific Psychiatry, and show that it is not.

The web site of the Council for Secular Humanism states: "Secular humanists reject authoritarian beliefs. They affirm that we must take responsibility for our own lives and the communities and world in which we live. Secular humanism emphasizes reason and scientific inquiry, individual freedom and responsibility, human values and compassion, and the need for tolerance and cooperation"[1].

The term "psychiatry" refers to both the principles and practices of this ostensibly medical specialty. It is necessary to emphasize at the outset that, unlike typical medical practices based on consent, typical psychiatric practices rest on coercion. In a free society, most social relations between adults are consensual. Consensual relations – in business, medicine, religion, and psychiatry – pose no special legal or political problems. In contrast, coercive relations – one person authorized to use the power of the state to compel another person to do or abstain from an action of his choice – are inherently political and morally problematic. In my following remarks I address only those relations between psychiatrists and patients that are actually or potentially coercive. In the prevailing legal and political climate (especially in the United States but by no means there alone), most psychiatric practices fall into this category.

It is not only the power but also the duty to coerce mental patients – to protect them from themselves and to protect society from them – that has always set psychiatrists apart from other medical practitioners. This is more true today than ever, but it is less obvious because it is better concealed.

When I was a medical student in Cincinnati in the early 1940s, there were no voluntary patients in Ohio state mental hospitals. A person could no more gain admission to a state mental hospital voluntarily than he could gain admission to a prison voluntarily. Individuals civilly committed to state mental hospitals were considered legally incompetent. In the old days of asylum psychiatry, the connection between mental illness and legal incompetence was unambiguous. If a person was mad enough to merit confinement in a madhouse, then he was incompetent. If he was not so confined, he was competent and safe from psychiatric coercion.

In the aftermath of World War II, partly as a result of the Nazi practice of exterminating mental patients, American social attitudes toward psychiatry and mental hospitals began to change. Erving Goffman's book, *Asylums*, and my book, *The Myth of Mental Illness*, both published in 1961, challenged the moral and legal legitimacy of psychiatric coercions, exemplified by involuntary confinement in a mental hospital [2]. Journalists compared state mental hospitals to concentration camps and called them "snake pits".

At this critical moment, so-called "psychiatric drugs" miraculously appeared. Politicians and the public quickly accepted the psychiatrists' claim that mental illnesses are brain diseases and that neuroleptic drugs are effective treatments for such diseases. Politicians and mental health professionals used this fiction as a peg on which to hang the complexly motivated program of emptying the state mental hospitals, misleadingly called "deinstitutionalization." In short, the three events characteristic of modern psychiatry – the "drug treatment" of mental illness, deinstitutionalization, and the conflation of mental illness and legal incompetence – occurred in tandem, each facilitating and supporting the others. At the same time, psychiatry – which had always been an arm of the coercive apparatus of the state – became more coercive and politicized. Politicians joined psychiatrists in authenticating and promoting the medical reality of "mental illnesses." The dual fictions – "mental illnesses are brain diseases effectively treated with drugs – became dogma, and deviation from it heresy. Herewith a few examples.

In 1999, President William Clinton declared: "Mental illness can be accurately diagnosed, successfully treated, just as physical illness" [3]. Tipper Gore, President Clinton's Mental Health Advisor, emphasized: "One of the most widely believed and most damaging myths is that mental illness is not a physical disease. Nothing could be further from the truth" [4]. First Lady Hillary Rodham Clinton explained: "The amygdala acts as a storehouse of emotional memories. And the memories it stores are especially vivid because they arrive in the amygdala with the neurochochemical and hormonal imprint that accompanies stress, anxiety, and other intense excitement. ... We must ... begin treating mental illness as the illness it is on a parity with other illnesses [5]. A *White House Fact Sheet on Myths and Facts About Mental Illness *declared: "Research in the last decade proves that mental illnesses are diagnosable disorders of the brain" [6].

With impressive naivete, then Surgeon General David Satcher, concluded: "Just as things go wrong with the heart and kidneys and liver, so things go wrong with the brain" [7]. The view that mental diseases stand in the same relation to brain diseases as, say, urinary problems stand in relation to kidney diseases is superficially attractive. The argument goes like this. The human body is a biological machine, composed of parts, called organs, such as the heart, the lung, and the liver. Each organ has a "natural function" and when this fails, we have a disease, such as coronary atherosclerosis, emphysema, hepatitis. If we define human problems as the symptoms of brain diseases, then they are brain diseases, even in the absence of any medically ascertainable evidence of brain disease. We can then treat mental diseases as if they were brain diseases.

The error in this reasoning is that if we add up all our body parts, the sum is obviously greater than its parts combined. A living human being is not merely a collection of organs, tissues, and cells; he is a person or moral agent. At this point the materialist-scientific approach to understanding and remedying its malfunctions breaks down. The pancreas may be said to have a natural function. But what is the natural function of the person? Theists and atheists have lungs and livers so similar that one may be transplanted into the body of another without altering his personal identity; but their beliefs and habits differ so profoundly that they often find it difficult or impossible to live with one another.

The truth is that the treatment of so-called mental diseases is no more successful today than it was in the past. Deinstitutionalization did not liberate mental patients. Some state mental hospitals inmates were transinstitutionalized, rehoused in parapsychiatric facilities, such as group homes and nursing homes. Others were imprisoned for offences they were prone to commit, transforming prisons into the nation's largest mental hospitals. Still others became "street persons", living off their social security disability benefits. Most idle, indigent, unwanted persons continue to be incarcerated in mental hospitals – intermittently, committed several times a year, instead of once for decades [8].

Most importantly, the powers of courts and mental health professionals were vastly expanded. Before World War II, they could control and forcibly "treat" only persons housed in mental hospitals. Armed with "outpatient commitment" laws, judges and psychiatrists can now control and forcibly "treat" persons living in the community.

The introduction of neuroleptic drugs into psychiatry created the illusion that mental illnesses, like medical illnesses, were "treatable" with drugs. Doubt about the benefits of long term mental hospitalization was replaced by confidence in the effectiveness of outpatient chemotherapy for mental illness. This development greatly enlarged the number of persons classified as mentally ill, contributed to the false belief that legal competence is a psychiatric issue, and further confused the legal relations between psychiatrist and mental patient.

Today, a person whose behavior is socially deviant – especially if he has once been a "mental patient" – risks being considered incompetent. For example, if such a person kills himself or someone else, then, after the fact and simply because of his act, he is considered incompetent and his psychiatrist's treatment of him is likely to be judged to be "medically negligent": regarded as the patient's guardian, the psychiatrist is considered to have failed to fulfil his "duty to protect" his ward or "third parties" endangered by his ward. None of this was true as recently as the 1960s [9].

To conduct arm-length relations with individuals we do not know, we must make certain presumptions about them. The automobile dealer presumes that his customer is legally competent and responsible for his purchase. The physician whose patient complains of blood in his stool presumes that the patient has a disease. The Anglo-American legal system presumes that a person accused of a crime is innocent until proven guilty, and competent until proven incompetent.

We are proud of our criminal justice system because it protects the accused from the power of the state, a power we distrust because its avowed aim is to harm the individual. Similarly, we are also proud of our mental health system, because it protects the mentally ill person from the dangers he poses to himself and others, a power we trust because its avowed aim is "therapeutic," not punitive.

Difficulties arise, however, once the power of the state to "help" goes beyond offering services (or money) and, instead, the state makes use of coercion. The justification for psychiatric coercion is further weakened by resting the requirement for commitment on "mental illness" and "dangerousness". There are no objective criteria for either mental illness or dangerousness. Thus, psychiatric determinations and declarations of their presence or absence are essentially oracular and rhetorical. Nevertheless, they fulfil a very important function: they instruct the listener to assume a desired attitude toward the "patient" [10].

Characterizing a door as brown or white is descriptive. Characterizing it as needing to be opened or closed is dispositive. Descriptive characterizations can be proved or disproved. Dispositive characterizations cannot, they can only be obeyed or disobeyed. The difference between the situation of the person accused of a crime and the situation of the person accused of mental illness is illuminating. The defendant has a right to deny his crime and disagree with his accusers. His insistence on his innocence is not interpreted as evidence of his guilt. The person diagnosed as mentally ill loses this right. His disagreement with the psychiatrist is interpreted as "lack of insight into his illness" or "denial of his illness". His insistence on his sanity is interpreted as evidence of his insanity.

Psychiatrists use the term "competent" as if they were identifying a "mental condition" *in *the designated person. That is why courts request the psychiatrist to examine defendants for competence, as if they were looking for and finding, or not finding, certain facts. Psychiatric "findings," however, especially in a forensic setting, are not facts but recommendations for a course of action toward the defendant.

Ironically, it is precisely because the American system of criminal justice is so intensely concerned with protecting innocent persons from punishment that it is especially vulnerable to corruption by excuses couched in terms of psychiatric disabilities and coercions justified as psychiatric treatments. The root of the problem lies largely in the concepts of mental illness and dangerousness, and partly in the doctrine of *mens rea*, sound mind.

The legal doctrine of mens rea, sound mind, which holds that unlawful behavior constitutes a crime only if it is committed by an actor who possesses a "guilty mind" – that is, whose "mind" can be held responsible (because it knows right from wrong), also works to strip the person incriminated as mentally ill of his rights. Since the Middle Ages and before, insane persons – perceived as similar to "wild beasts" – have been regarded as lacking mens rea. This is why "infants, idiots, and the insane" – in John Locke's famous phrase, repeated unchanged ever since – are not prosecuted or punished by the criminal law, but instead are restrained, as minors and as mad, by family courts and mental health laws.

Treating mentally ill persons as if they were like children fails to take into account the many obvious differences between them. Minority is an objectively defined (chronological) condition and a legal status. Mental illness is neither. Children are, by definition, under tutelage. Few mental patients are under tutelage and those that are, are in that status not because they are mentally ill but because they are declared to be legally incompetent.

I maintain that "mental illness" is not something the patient has, it is something he is. The modern psychiatrist is likely to view Lady Macbeth as insane, the victim of manic depressive psychosis, an illness that renders her not responsible for her crimes. Shakespeare viewed her as "Not so sick...as troubled with thick coming fancies," for which "more she needs the divine than the physician" [11]. Ironically, today's Lady Macbeths, female and male alike, receive the ministrations of divines, albeit they are called "medical doctors" and are licensed physicians (or pseudo-physicians, called "mental health professionals"). I interpret this as a symptom of the transformation of the theocratic state into the therapeutic state.

In *Democracy in America*, Alexis de Tocqueville warned: "The species of oppression by which democratic nations are menaced is unlike anything that ever before existed in the world ... Above this race of men [incessantly endeavoring to procure their petty and paltry pleasures] stands an immense and tutelary power, which takes upon itself alone to secure their gratifications and to watch over their fate. ... For their happiness such a government willingly labors ... provides for their security ... facilitates their pleasures, manages their principal concerns..." [12].

Did Tocqueville foresee the coming of pharmacracy, that is, government informed and legitimized not by gods (theocracy), social position (aristocracy), or popular sovereignty (democracy), but by medicine and its ideology that tends to define human problems as "diseases," curable by coercions defined as "treatments"? Evidence for the medicalization of every kind of undesirable behavior abounds.

I first proposed the term "pharmacracy" in 1974, to complete a triad of phrases to identify that we are in the process of a profound cultural transformation [13]. Prior to World War II, the American system of social controls rested on Christian moral values and was enforced by a judicial apparatus based on English common law, the Constitution, and the rule of law. Since then, our system of social controls has become increasingly dependent on the principles of a politicized medicine, and has been legitimized and enforced by a complex state apparatus that commingles the principles and practices of paternalistic "therapy," punitive psychiatry, collectivistic public health, and the criminal justice system.

To articulate this insight, in 1960 I proposed the phrase "myth of mental illness." The term was intended to lay bare that the phenomena we so label are neither mental nor illnesses, and that the measures used to remedy them are not treatments but efforts to tranquilize, pacify, and subdue the disturbing person [14]. In 1963, I proposed the term "therapeutic state" to identify the transformation of our dominant political ideology from a democratic welfare state legitimized by the rule of law into an autocratic therapeutic state legitimized by psychiatry as a branch of medicine.

Finally, in 1974, in Ceremonial Chemistry, I wrote: "Inasmuch as we have words to describe medicine as a healing art, but have none to describe it as a method of social control or political rule, we must first give it a name. I propose that we call it pharmacracy, from the Greek roots pharmakon, for 'medicine' or 'drug,' and kratein, for 'to rule' or 'to control.' ... As theocracy is rule by God or priests, and democracy is rule by the people or the majority, so pharmacracy is rule by medicine or physicians" [15]. In a theocracy, people perceive all manner of human problems as religious in nature, susceptible to religious remedies. Similarly, in a pharmacracy people perceive all manner of human problems as medical in nature, susceptible to medical remedies.

Perceptive writers – for example, Samuel Butler, Aldous Huxley, C. S. Lewis, and Adolfo Bioy Casares – foresaw this trend and described some of the features of the therapeutic state and the pharmacratic controls that characterize it. In his satirical dystopia, *Erewhon *(1872), Butler wrote: "As I have already said, these [persons we regard as criminals], though not judicially punishable, are recognized as requiring correction. Accordingly, there exists a class of men trained in soul-craft, whom they call straighteners, as nearly as I can translate a word which literally means "one who bends back the crooked." ... Indeed, the straighteners have gone so far as to give names from the hypothetical language (as taught at the College of Unreason) to all known forms of mental indisposition, and to classify them according to a system of their own, which, though I could not understand it..." [16].

In *Erewhon Revisited *(1901), Butler presciently debunked the now virtually unchallenged poppycock that psychotropic drugs "cure" bad behavior. He wrote: "No more swearing. No more bad language of any kind. A lamb-like temper ensured in about twenty minutes, by a single does of one of our spiritual indigestion tabloids. In cases of all the more ordinary moral ailments, from simple lying to homicidal mania, in cases again of tendency to hatred, malice, and uncharitableness ... our spiritual indigestion tabloids will afford unfailing and immediate relief" [17].

Toward the end of *Brave New World *(1932) – the world in which all conflicts and discomforts have been eliminated – the human remnant Huxley calls the Savage, and the dictator whom Huxley calls the Controller, Mustapha Mond, engage in the following dialogue: "We prefer to do things comfortably" [said the Controller]./"But I don't want comfort. I want God, I want poetry, I want real danger, I want freedom, I want goodness, I want sin."/"In fact, said Mustapha Mond, "you're claiming the right to be unhappy."/

"All right, then," said the Savage defiantly, "I'm claiming the right to be unhappy" [18].

More recently, C. S. Lewis (1953/1970) warned: "Of all the tyrannies a tyranny sincerely exercised for the good of the victims may be the most oppressive ... To be 'cured' against one's will and cured of states which we may not even regard as disease is to be put on a level with those who have not yet reached the age of reason or those who never will; to be classed with infants, imbeciles, and domestic animals. For if crime and disease are to be regarded as the same thing, it follows that any state of mind which our masters choose to call 'disease' can be treated as a crime; and compulsorily cured. Even if the treatment is painful, even if it is life-long, even if it is fatal, that will be only a regrettable accident; the intention was purely therapeutic [19].

In our own day (1986), Argentinean novelist Adolfo Bioy Casares observed: "Well then, maybe it would be worth mentioning the three periods of history. When man believed that happiness was dependent upon God, he killed for religious reasons. When he believed that happiness was dependent upon the form of government, he killed for political reasons. ... After dreams that were too long, true nightmares. ... we arrived at the present period of history. Man woke up, discovered that which we always knew, that happiness is dependent upon health, and began to kill for therapeutic reasons. ... It is medicine that has come to replace both religion and politics in our time" [20].

I have asserted that Secular Humanism is incompatible with the principles and practices of psychiatry. However, I do not speak for Secular Humanism. Those who do must decide whether that decision is well-founded or not. I ask only that the decision-makers keep in mind two things: 1) The paradigmatic practices of psychiatry are civil commitment and insanity defense – that is, depriving innocent persons of liberty and excusing guilty persons of their crimes – and that the consequences of both are confinement in institutions ostensibly devoted to the treatment of mental diseases. 2) *Black's Law Dictionary *states: "Every confinement of the person is an 'imprisonment,' whether it be in a common prison, or in private house, or in the stocks, or even by forcibly detaining one in the public streets" [21].
